# Trial sequential analysis suggested the potential overestimated effect of carbonic anhydrase inhibitor for respiratory failure and metabolic alkalosis

**DOI:** 10.1186/s13054-019-2384-y

**Published:** 2019-04-24

**Authors:** Meng-Si Luo, Hui-Zi Li, Guan-Jiang Huang, Lun Wu

**Affiliations:** 1 Department of Anesthesiology, Zhongshan Hospital of Traditional Chinese Medicine, Affiliated to Guangzhou University of Chinese Medicine, 3 Kangxin Road, Zhongshan, 528400 Guangdong China; 2 Department of Orthopaedics, The Fifth Affiliated Hospital of Sun Yat-Sen University, 52 Meihua East Road, Xiangzhou District, Zhuhai, 519000 Guangdong China; 3 Department of Otorhinolaryngology, The Second Affiliated Hospital, School of Medicine, Zhejiang University, 88 Jiefang Road, Hangzhou, 310009 Zhejiang China

Meta-analyses of randomized controlled trials (RCTs) used to be considered as the optimum evidence to guide clinical practices. Generally, a high-quality meta-analysis with conclusive information should meet the minimum requirements of a well-conducted RCT, which includes prospective protocol development, limitation of bias, and adequate sample size [1]. Conversely, meta-analyses based on limited RCTs may trigger the potential overestimation of the authentic intervention effect owing to weak statistical power [1]. More interestingly, increasing studies indicated that pooled results with false positive were frequently existed in published meta-analyses including many Cochrane ones [2, 3].

Trial sequential analysis (TSA) was introduced to monitor potential random error, false positive, and false negative in meta-analyses of RCTs [3]. Moreover, it was recommended that TSA should be performed to assess the “imprecision” of outcomes of interest in the Grading of Recommendations Assessment, Development and Evaluation (GRADE) [4]. A recent meta-analysis indicated that carbonic anhydrase inhibitor (CAI) may have a positive effect on respiratory failure and metabolic alkalosis [5]. Considering that limited trials with small information size included in the study, we assumed that the effect of CAI for respiratory failure and metabolic alkalosis may be overestimated. Subsequently, we performed TSA for one of outcomes (i.e. PaCO_2_) with the most included RCTs to estimate whether the evidence is enough reliable and credible. TSA in Fig. 1 showed that the cumulative *Z*-curve did not cross the trial sequential monitoring boundary for benefit and the required information size boundary, which suggested that the current evidence (the positive effect of CAI on PaCO_2_) was inconclusive. In addition, TSA on PaCO_2_ showed that the required information size (347 patients) is not reached due to weak statistical power. So, the effects of CAI therapy for patients with respiratory failure and metabolic alkalosis may very likely be overrated.

**Fig. 1:**
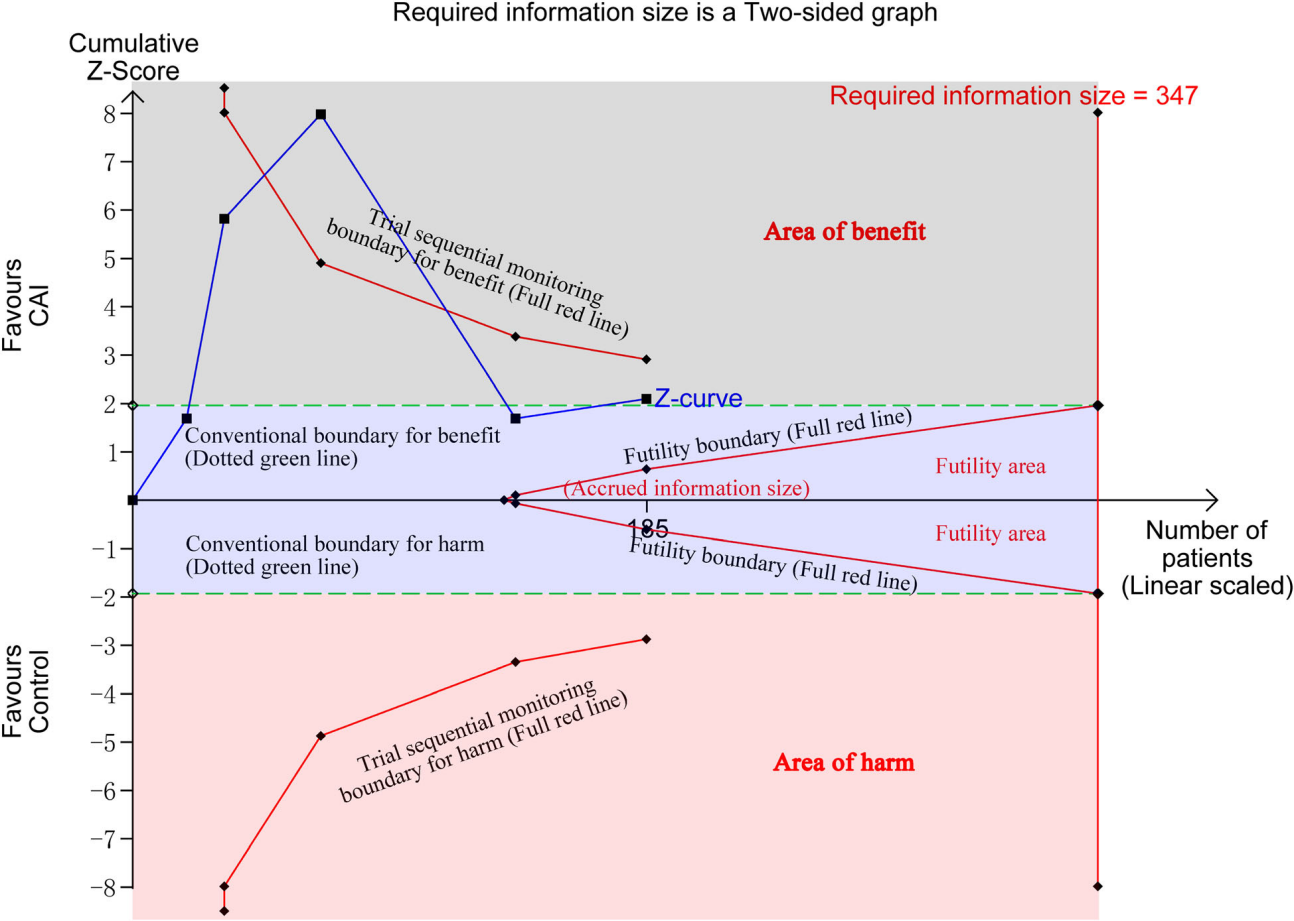
Trial sequential analysis (TSA) of 5 trials testing the effect of CAI therapy on PaCO_2_ in patients with respiratory failure and metabolic alkalosis. TSA of 5 trials (black square fill icons) showing that the line of cumulative *Z*-curve crossed the conventional boundary for benefit, but not the lines of the trial sequential monitoring boundary for benefit and required information size (RIS), which establish inconclusive evidence and suggest that further trials are needed. (*X*-axis, number of patients; *Y*-axis, cumulative *Z*-score; horizontal green dotted lines, conventional boundaries for benefit or harm; sloping full red lines with black square fill icons, trial sequential monitoring boundaries for benefit or harm; full blue line with black square fill icons, *Z*-curve; vertical red full line, required information size boundary)

Collectively, for meta-analyses of RCTs with limited information size, TSA is a good choice to monitor the potential overestimation of the overall pooled effect. Furthermore, it is worthwhile to further discussion whether TSA should be routinely performed in meta-analyses of RCTs.

## Abbreviations

CAI: Carbonic anhydrase inhibitor
GRADE: Grading of Recommendations Assessment, Development and Evaluation
RCT: Randomized clinical trial
TSA: Trial sequential analysis
