# Supplementary material for: Trial sequential analysis suggested the potential overestimated effect of carbonic anhydrase inhibitor for respiratory failure and metabolic alkalosis
Source: Crit Care. 2019 Apr 24;23:138. doi: 10.1186/s13054-019-2384-y (PMC6480612; doi:10.1186/s13054-019-2384-y)
Supplement: Related texts

# Reply: Authors' response

**Authors:** Bassem Y. Tanios, Pierre K. Bou-Khalil, Samir S. Mallat, Elie A. Akl

We thank Meng-Si Luo et al. for their interest in our work, and for highlighting one the limitations of the literature in this field, namely the limited number of published randomized controlled trials (RCTS). In our conclusion, we do acknowledge this important limitation and the need for larger, well designed, and RCTs addressing clinically important outcomes such as mortality, duration of hospital stay, and duration of mechanical ventilation. [5]

Meng-Si Luo et al. propose the use of Trial Sequential Analysis (TSA) to assess the ‘imprecision’ of outcomes. In a recent expert panel consensus statement, the Cochrane scientific committee recommended against the use of sequential methods for the main analysis, or to draw main conclusions. The statement encourages authors to interpret evidence based on the estimated magnitude of the effect of intervention and its uncertainty (usually quantified using a confidence interval), rather than focusing primarily on the rejection of the null hypothesis of no treatment effect (like that used by Meng-Si Luo et al. in their TSA) [6].

Our approach was consistent with the recent Cochrane recommendations, as we did not draw binary interpretation of the effect estimate as “significant” or “non-significant”. Instead, we presented our results using a confidence interval and assessed heterogeneity using the I2 statistic, then used the GRADE methodology to rate the certainty of evidence. In applying GRADE, we judged imprecision as a reason for rating down the certainty of evidence for all the clinically important outcomes. [6, 7] If we were to analyze the certainty of evidence for the change in PaCO2 using the GRADE methodology, we would have similarly rated down the certainly of evidence for imprecision (and further down due to significant heterogeneity resulting in a low certainty evidence). Indeed, our meta-analysis found a mean reduction of - 4.98 mmHg in the carbonic anhydrase inhibitors group (95% CI -9.66 to -0.3; I2 = 95%). The confidence interval includes both values indicating a clinically significant benefit and values indicating no effect [8].

One of the main reasons Cochrane adopted the above recommendation is to “support the decision maker and end user by providing the best and latest evidence, but that interpretation of that evidence should be left to the user to make within their own context.” We are happy our approach was consistent with that recommendation given the aim of our review is to support clinicians providing care to critically ill patients.
